# A Review of Risk Factors and Predictors for Hemorrhagic Transformation in Patients with Acute Ischemic Stroke

**DOI:** 10.1155/2021/4244267

**Published:** 2021-12-06

**Authors:** Sneha E. Thomas, Noorine Plumber, Priyanka Venkatapathappa, Vasavi Gorantla

**Affiliations:** ^1^Department of Internal Medicine, University of Maryland Medical Center, Baltimore, USA; ^2^Department of Anatomical Sciences, St. George's University School of Medicine, Grenada W.I; ^3^University Health Services, St. George's University School of Medicine, Grenada W.I

## Abstract

Acute ischemic strokes (AIS) and hemorrhagic strokes lead to disabling neuropsychiatric and cognitive deficits. A serious and fatal complication of AIS is the occurrence of hemorrhagic transformation (HT). HT is cerebral bleeding that occurs after an ischemic event in the infarcted areas. This review summarises how specific risk factors such as demographic factors like age, gender, and race/ethnicity, comorbidities including essential hypertension, atrial fibrillation, diabetes mellitus, congestive heart failure, and ischemic heart disease along with predictors like higher NIHSS score, larger infarction size, cardioembolic strokes, systolic blood pressure/pulse pressure variability, higher plasma glucose levels, and higher body temperature during ischemic event, lower low-density lipoprotein and total cholesterol, early ischemic changes on imaging modalities, and some rare causes make an individual more susceptible to developing HT. We also discuss few other risk factors such as the role of blood-brain barrier, increased arterial stiffness, and globulin levels in patients postreperfusion using thrombolysis and mechanical thrombectomy. In addition, we discuss the implications of dual antiplatelet therapy and the length of treatment in reference to the incidence of developing HT. Current research into inflammatory mediators and biomarkers such as Cyclooxygenase-2, matrix metalloproteinases, and soluble ST2 and their potential role as treatment options for HT is also briefly discussed. Finally, this review calls for more research into use of dual antiplatelet and the timing of antiplatelet and anticoagulant use in reference to hemorrhagic transformation.

## 1. Introduction

Strokes are the second leading cause of death worldwide and the fifth leading cause of death in the United States [1–4]. Strokes affect approximately 14 million people each year, with 5.5 million dying and another 5 million suffering disabling complications [4]. Ischemic strokes account for 85 percent of all strokes, while hemorrhagic strokes account for the remaining 15%, both of which can result in life-threatening complications including death. The widespread prevalence of strokes globally emphasizes the need for researchers and healthcare providers to understand the risk factors, complications, and optimize treatment regimens. Strokes, in general, can occur for a variety of reasons, including modifiable risk factors like diet, exercise, and environmental conditions, as well as nonmodifiable risk factors like genetic predisposition, age, and gender [5, 6]. Stroke risk may also be increased by the presence of other comorbidities such as hypertension, atherosclerosis, atrial fibrillation, and diabetes mellitus.

Ischemic strokes arise from severe occlusion and reduction in cerebral perfusion, which then results in cerebral hypoxia, irreversible damage, and necrosis of brain tissue [4]. Additionally, conditions such as hemodynamic instability, inflammatory cascades, and permeability of the blood-brain barrier along with infiltration of glial cells and leukocytes can further contribute to narrowing of cerebral vasculature and result in ischemic strokes. The severity of an acute ischemic stroke (AIS) is assessed and categorized using the National Institutes of Health Stroke Scale (NIHSS) in which 0 = no stroke symptoms, 1 − 4 = minor stroke, 5 − 15 = moderate stroke, 16–20 = moderate/severe stroke, and 21–42 = severe stroke [7]. Imaging studies such as computed tomography (CT) and magnetic resonance imaging (MRI) with and without contrast are used to identify the presence and severity of either hemorrhagic or ischemic stroke to incorporate appropriate treatment plans for patients. Hemorrhagic strokes have limited treatment options as profuse cerebral bleeding may lead to various complications including herniation and fatalities. Therefore, hemorrhagic strokes are associated with increased mortality rates due to its severity and limited management and treatment options available.

One of the major, and potentially life threatening, complications of AIS is hemorrhagic transformation (HT), also known as ischemia-related hemorrhage. The pathophysiological mechanism of HT is thought to be due to necrosis and disruption of cerebral metabolism resulting from cerebral ischemia caused by arterial occlusions. The sodium-potassium ATP pumps are disrupted as a result of the hypoxic environment, which causes a decrease in Adenosine triphosphate (ATP) concentrations [8–10]. The intracellular accumulation of Na+ causes cytotoxic edema in the brain leading to blood-brain barrier (BBB) disruption and the neurovascular unit composed of neurons, glia, endothelial cells, vascular smooth muscle, and immune cells [11–13]. Immune-mediated inflammatory response by activated neutrophils, reactive oxygen species produced by monocytes, and various types of matrix metalloproteinases contribute to this BBB disruption [13–15]. Other immune cells such as microglia and astrocytes along with endothelial cells also play major roles in the inflammatory response [13–15]. Extravasation of blood and infiltration of inflammatory mediators such as cytokines and metalloproteinases into cerebral tissue are promoted by further damage to the endothelial layer of capillaries and increased permeability of the blood-brain barrier, resulting in HT [12, 16, 17].

Hemorrhagic transformation can occur as spectrum but is commonly subdivided into two major subtypes, hemorrhagic infarction (HI) from petechial hemorrhages and parenchymal hematoma (PH) [8, 12, 18, 19]. A study by Larrue et al. classified HT into hemorrhagic infarction (HI), parenchymal hemorrhage (PH), and symptomatic intracranial hemorrhage (SICH) [20]. HI and PI were further divided into HI1, small petechiae along the margins of the infarct, and HI2, confluent petechiae within the infarcted area but no space-occupying effect, PH1, as blood clots in ≤30% of the infarcted area with some slight space-occupying effect; and PH2, as blood clots in >30% of the infarcted area with a substantial space-occupying effect. Symptomatic intracranial hemorrhage (SICH) was termed if there was a clinical deterioration leading to NIHSS score of ≥4 points and if the hemorrhage was likely to be the cause of the clinical deterioration [20]. Regardless of slight variations in definition, in perfusion studies, HI presents as heterogeneous and occupies a portion of the infarcted area, whereas PH shows as a dense hematoma and more homogenous [21–25]. Misdiagnosis and/or mislabeling of HT as intracranial hemorrhage, ICH, due to delayed findings of HT on CT imaging is also seen in the literature and is something that has been discussed by few investigators in the past [18, 26]. HT can occur spontaneously after physiologic reperfusion as the natural course of AIS but can also occur after thrombolytic therapy such as tissue plasminogen activator (tPA or rtPA) and/or mechanical reperfusion therapies such as thrombectomy [27]. It affects 10-40% of people who have had an ischemic stroke. In autopsy studies, the incidence of spontaneous HT ranges from 38 to 71% while it ranges from 13-46% on CT studies [21]. The incidence of symptomatic HT ranges from 0.6 to 20% [21, 24, 28, 29]. In a prospective study that consisted of 407 patients with ischemic strokes, researchers analyzed the frequency of spontaneous HT occurrence in relation to the infarcted area using CT and MRI techniques. Tan et al. found that 50 patients (12.3%) suffered a spontaneous HT, with 66% of cases being HI and 34% being PH [13]. HT can present as asymptomatic or symptomatic, with severe neurological deficits due to profuse bleeding in the infarcted areas.

## 2. Timing of Hemorrhagic Transformation

Although HT can occur at various timeframes, it typically occurs within the first week after AIS. A retrospective cohort study performed by Muscari et al. showed a median of 6 days with a range of 1-27 days [30]. HT is, sometimes, further divided into early HT, <48 hours, and late HT, >48 hours, in literature [31–33]. Some animal studies have used <18-24 hours as early HT vs. >18-24 hours as late HT [14]. Specific pathophysiology behind early and late HT is slightly different: early HT occurs due to reperfusion due to leptomeningeal anastomoses as a result of clot movement while late HT occurs as a result of increased vascular permeability and increased blood flow after reduction in cerebral edema [31, 32, 34–36].

## 3. Risk Factors and Predictors Associated with Hemorrhagic Transformation

The presence of several similar and different risk factors that increase the risk of strokes and HT has been discovered in recent studies. In terms of strokes in general, unhealthy environmental and lifestyle choices such as smoking, alcohol and drug use, limited physical activity, and irregular sleeping patterns are all associated with higher stroke incidences and worsening morbidity and mortality rates [5]. Incorporating effective lifestyle and medical interventions can significantly decrease the risk of stroke occurrence. Age, sex, ethnicity, diabetes, hyperlipidemia, atrial fibrillation, previous stroke, and onset to treatment time are all nonmodifiable risk factors that play a significant role in the development of strokes. Predictably, many of the risk factors for stroke are the same for hemorrhagic transformation as well. Understanding these modifiable and nonmodifiable risk factors and evaluating predictors in depth is crucial to improve patient outcomes and quality of life. Below, we review some of the major risk factors (conditions/factors present in the patient) and predictors (characteristics or complications that occur during the AIS event) associated with HT.

## 4. Demographic Factors: Age, Race, and Gender

Strokes, according to Boehme et al., are more common in the elderly population, though the incidence of stroke in the 20–54 year age group has increased recently, from 12.9 percent in 1993 to 18.5 percent in 2005 [5]. Howard et al. found that the incidence of cardiovascular complications such as hypertension, diabetes mellitus, dyslipidemia, and atrial fibrillation was higher in older adults over the course of a 10-year follow-up of 10,801 adults in a longitudinal race-related cohort study [37]. Similarly, HT is also more commonly seen with advanced age. A retrospective study by Pande et al. showed older age, median age of 68.4 vs. 65.4 with *p* value of 0.001, was associated with significant risk for HT [31]. This is especially true for elderly patients over the age of 80 treated with thrombolytic therapy such as r-tPA [20, 38–40].

In comparison to Caucasians, African Americans and Hispanic Americans have twice the risk of stroke and have a higher morbidity and mortality rate [5]. African Americans also had a higher rate of hypertension, diabetes, and dyslipidemia, but a lower rate of atrial fibrillation [37]. This suggests that cardiovascular complications in older adults and certain races may lead to significant atherosclerosis, vasculature damage, and stroke development [41]. Strokes are more common in women than in men [42]. This is thought to be due to factors such as longer life expectancy in women, contraceptive use, and pregnancy-related complications. However, the overall risk for HT is similar between men and women, although few studies have shown higher risk in men [43].

## 5. Cardioembolic Stroke: History of Atrial Fibrillation, Ischemic Heart Disease, and Congestive Heart Failure

Atrial fibrillation is a notable risk factor for HT as it can result in cardioembolic cerebral infarction [44, 45]. Tu et al. examined the association between atrial fibrillation and worsening neurological impairment in a trial of 101 ischemic stroke patients [44]. In this study, researchers found that patients with a history of atrial fibrillation had more severe cerebral hypoperfusion (*p* = 0.02) along with more frequent and severe HT in comparison to patients without atrial fibrillation [44]. A prospective multicenter study by Paciaroni et al. looked at 1125 patients where 98 patients (8.7%) had HT, 62 (5.5%) had hemorrhagic infarction, and 36 (3.2%) parenchymal hematoma. HT was associated with the presence of atrial fibrillation (29/98, 29.6% versus 189/1027, 18.4%; *p* = 0.01) and cardioembolic stroke (51/98, 52.0% versus 249/1027, 24.2%; *p* < 0.0001), especially parenchymal hematoma [46]. Some studies have also noted that the presence and extent of white matter lesion in patients with atrial fibrillation also place them at an increased risk of HT [47].

Cardioembolic stroke in itself is a predictor for spontaneous HT, especially in patient not treated with thrombolytic agents [48]. Studies by Choi et al., Bayramoglu et al., Paciaroni et al., and Wen et al. have all noted that cardioembolic origin is a frequent predictor for HT [43, 46, 48–50]. Strokes attributable to cardioembolism were also an independent predictor for HT (OR 2.36; 95% CI 1.44 to 3.68) [46].

History of congestive heart failure (CHF) is also associated with increased risk of HT (OR 1.49; 95% CI 1.05-2.20; *p* = 0.02) [47, 51]. Atherosclerosis, interestingly, was not associated with increased risk for HT in a study by Celik et al., although the risk to develop AIS is significantly higher in this group of patients [48, 52]. Prior history of ischemic heart disease and cerebral vascular disease also increase the risk of HT [53].

Anticoagulation in atrial fibrillation and the timing of initiation anticoagulation in AIS is a very controversial topic and area that is being actively studied currently. Anticoagulants such as vitamin K antagonist or novel oral anticoagulants (NOACs) are often indicated in patients with cardioembolic stroke to reduce the risk of recurrent ischemic stroke. However, there is an increased risk of HT associated with anticoagulant use [31, 39, 51, 54–56]. According to AHA/ASA guidelines, starting or restarting oral anticoagulants within 4-14 days of ischemic stroke is considered reasonable and a later start is to be considered in patients with HT [57]. Results of currently ongoing studies and new randomized control trials are needed to optimize the anticoagulation management in AIS and HT.

## 6. Hypertension and Systolic Blood Pressure Variability

Hypertension is strongly associated with increased risk of strokes, and studies have shown that reducing systolic blood pressure by 5-6 mmHg reduced the relative risk of stroke by 42% [5].

There is some controversy on the role of hypertension history as a risk factor in HT. Chronic hypertension, certainly, affects cerebral vasculature, lumen diameter, impair endothelial function, and increase BBB permeability [13, 58]. It can impair collateral circulation, reducing capacity to maintain adequate oxygenation when a cerebral artery occlusion occurs. Systolic blood pressure (SBP) and SBP variability, on the other hand, have received tremendous importance in the recent years as there is rising evidence showing an association with severe HT. Systolic blood pressure (SBP) variability is being closely monitored and studied lately especially in the setting of thrombolytics and is often considered as predictor for HT during the AIS [20]. Elevated systolic blood pressure increases the risk of HT via multiple mechanisms, similar to chronic hypertension, such as its effects on the vasculature and vascular remodeling affecting collateral circulation, autoregulation, and inflammatory response leading to disruption in BBB [13, 58, 59]. Liu et al. investigated the relationship between hour-to-hour BP variability and HT after IV thrombolytic therapy. In this study, high SBP variability during the first 6 hours after thrombolytic therapy was related with severe HTs [60]. In animal models, decreasing blood pressure prior to stroke was associated with decreased risk of HT [61].

Although current guidelines suggest that systolic blood pressure (SBP) of 180 mmHg can be used as an effective upper limit, Silverman et al. suggest that maintaining blood pressure in an optimized range personalized to each patient may result in decreased occurrence of HT [62]. Improved stroke outcomes are seen when BP is maintained between 140-180 mmHg [13, 63]. This study further indicates that increasing the SBP above the upper limit of autoregulation, about 120 mmHg results in hyperperfusion and increases the risk of HT [62].

The ENCHANTED trial, by Anderson et al., also compared standard SBP control (SBP < 180 mmHg) with tight blood pressure control (SBP target of 130–140 mmHg). In this trial, although intensive control of BP was noted to be safe and associated with a decreased incidence in HT (18.7% to 14.8% with *p* = 0.013), no improvement in 90-day Modified Rankin Scale was seen [64]. Close monitoring of each patients hemodynamics may allow a tailored and individualized treatment plan in these specific clinical settings.

## 7. Lipid Profile

Several studies also suggest that low levels of total cholesterol (TC) and low-density lipoprotein (LDL) levels are associated with HT. Yang et al. analyzed lipid profiles of 348 AIS patients and HT was noted in 35 patients. In comparison to the non-HT groups, the HT group had low levels of total cholesterol and LDL [45]. Paciaroni et al. noted that a low LDL-cholesterol level on admission (median 107.20 ± 30.3 mg/dL vs. 115.60 ± 35.4 mg/dL; *p* = 0.02) was associated with HT [46]. In another study by Wang et al., researchers analyzed lipid profiles of 1239 AIS patients 24 h after admission. The ratio of low-density lipoprotein to high-density lipoprotein levels was examined. Wang et al. found significantly lower LDL/HDL levels (*p* = 0.02) in the 10.9% of patients that developed in HT in comparison to those without HT [65]. Similarly, Ocek et al. noted serum LDL, triglycerides, and TC were significantly lower in patients with HT (*p* < 0.001) [48]. This suggests that the use of lipid-lowering medications may need dosage adjustments in some patients to reduce the incidence of HT. The relationship between HT and low LDL levels is controversial as statins help reduce LDL levels and result in better management of atherosclerosis [65]. Therefore, further research is indicated to examine the correlation between TC and LDL levels and the incidence of HT in AIS patients.

## 8. Diabetes Mellitus and Hyperglycemia

Like hypertension, diabetes mellitus is also an important risk factor for strokes and HTs [43].

Chronic diabetes mellitus contributes to a worse functional outcome in stroke patients compared to those without diabetes [66]. Hyperglycemia itself is also a strong predictor for HT occurrence [43, 45, 46]. Although the exact mechanism is unclear, multiple studies have shown that hyperglycemia on admission or persistent hyperglycemia during AIS is associated with HT [43, 66]. Desilles et al. conducted a study on hyperglycemic and normoglycemic rats by inducing a middle cerebral artery occlusion. They found that hyperglycemic rats had accelerated BBB damage and incomplete reperfusion despite being treated with recanalization [67]. They also noted that hyperglycemic rats had an exacerbated neuroinflammatory cascade that increased the incidence of hemorrhagic transformation in comparison to normoglycemic rats [67]. A meta-analysis by Wen et al. noted that in Chinese patients with AIS receiving thrombolytic therapy, diabetes was associated with increased HT [43]. A study Paciaroni et al. noted that hyperglycemia on admission predisposes to PH and an overall nonfavorable outcome at 3 months. He noted a linear relationship between PH and hyperglycemia with 2.1% rate of PH in patients with <110 mg/dl, 3.6% in patients with a level between 110 and 149 mg/dl, and 6.4% in patients with a level > 150 mg/dl [68].

## 9. NIHSS

Multiple studies have noted that high NIHSS is associated with HT [31, 69, 70]. A study by Paciaroni et al. noted that high NIHSS score on admission (median 14.0 versus 6.0; *p* < 0.0001) was associated with HT while Kablau et al. noted similar findings (mean NIHSS score 9.9 vs. 5.9; *p* = 0.003) [46]. Similar findings were also noted by others like Wen et al. and Ocek et al. [43, 48].

## 10. Size of Infarction

Another significant predictor of HT is the size of infarct. A massive cerebral infarction shows a positive correlation between the size of infarcted area and incidence of HT [38, 39, 41, 43, 46, 53]. A huge inflammatory response and edema associated with the massive cerebral infarct plays a role in the increased HT risk [24]. For instance, HT was noted to be inversely associated with lesions of less than 1.5 cm (3/98, 3.0% versus 475/1027, 46.2%; *p* < 0.0001) where large anterior stroke (53/98, 54.1% versus 131/1027, 12.7%; *p* < 0.0001) was associated with HT. Large lesions (OR 4.57, 95 CI 2.83 to 7.39) were also an independent predictor for HT overall [46]. In terms of location, middle cerebral artery (MCA) strokes are more commonly associated with HT [31]. Kablau et al. also noted that territorial infarction (88 vs. 58.8; *p* = 0.007) was associated development of HT [69].

### 10.1. Prolonged Ischemia

Studies have shown that prolonged ischemia with delayed reperfusion causes worse HT [71]. Reperfusion appears to occur immediately after the obstructing suture is removed from a cerebral artery in several mouse studies, but reperfusion timeframes and HT from stroke onset vary greatly in humans [71].

### 10.2. Collateral Circulation

Poor collateral circulation, which sustains the penumbra, results in faster expansion of infarct core, is associated with risk of HT [13, 24, 72].

### 10.3. Electrolyte Abnormalities

Certain electrolyte abnormalities such as hyperkalemia and hypomagnesemia are associated with HT. Magnesium, in fact, is considered an endogenous neuroprotective agent via its anti-inflammatory and antioxidation properties [73–75]. A retrospective study was conducted by Cheng et al. on 242 patients receiving thrombolysis, and the patient that developed HT was noted to have significantly lower serum magnesium levels than those without HT (0.81 ± 0.08 vs. 0.85 ± 0.08 mmol/L, *p* = 0.007) [73]. Pande et al. noted that higher potassium level (4.25 vs. 4.07; *p* = 0.007) was associated with significant risk of HT [31].

### 10.4. Other Factors

Cardiovascular complications and changes in cerebral vasculature occur from substances such as alcohol and tobacco. In a meta-analysis by Janket et al., it was noted that patients who had periodontal diseases had a 19% increased risk of developing cardiovascular complications such as stroke and heart disease [76]. Leukoaraiosis and leukoplakia are also associated with increased risk of HT which has been mentioned in literature previously [77, 78]. Oral leukoplakia presents as thick, white patches in the mouth and has been associated with prolonged smoking and alcohol use. Limiting the use of these substances can promote oral health and reduce the risk of cardiovascular and stroke occurrence [76].

Higher body temperature within the first 24 hours after AIS is another interesting predictor for hemorrhagic transformation [31]. A study conducted by Leira et al. on 229 patients with ischemic stroke untreated with rPA, higher body temperature during the first 24 h (36.9°C compared with 36.5°C) was associated with HT (*p* < 0.0001) [79].

### 10.5. Immune Mediators and Hemorrhagic Transformation

Finally, many inflammatory components and proteolytic enzymes may also play a role in HT.

Inflammatory markers such as leukocytes and C-reactive proteins were also elevated in patients that developed HT. Matrix metalloproteinase (MMP) 3, MMP-9, platelet-derived growth factor-CC, reactive oxygen species (ROS), and reactive nitrogen species (RNS) are all components of inflammatory response and have been implicated in BBB disruption [13]. MMP-9 is a potent proteolytic enzyme and the integrity of the BBB. Mechtouff et al. analyzed the effect of MMP-9 and the occurrence of hemorrhagic transformation [80]. Researchers examined the extent of infarct growth and levels of MMP-9 in AIS patients, and the study suggests that high levels of MMP-9 (*p* = 0.02) and delayed reperfusion were associated with increased infarct growth [80]. Novel research in which genetic deletions or downregulation of COX-2 and MMP-9 expression has shown reduced BBB damage and reduction in the risk of developing HT [14, 81]. ST2 is another novel member of toll-like receptor superfamily which plays a role in inflammatory signaling of helper T-cells and neuroinflammation. There is growing evidence that plasma sST2 is associated with increased risk of HT after adjusting for other traditional risk factors (*p* = 0.039) [82]. The reduction in proteolytic degradation and neuroinflammatory cascade may contribute to decreased BBB damage and add neuroprotection for vulnerable AIS patients. In order to implement similar gene therapies, further research is indicated to evaluate its benefits and efficacy.

### 10.6. Imaging Characteristics

Detecting early ischemic changes through noncontrast CT can provide significant diagnostic data. CT scans are the diagnostic tool of choice due to its ease of use, cost-effectiveness, and noninvasive imaging technique. Studies show that identifying these ischemic changes predict patient functional outcomes and clinical manifestations based on stroke severity. Mair et al. conducted a study in which 342 scans from 200 patients were analyzed to measure ischemic attenuation to determine stroke onset times. Researchers found that early detection of ischemic lesions is helpful in identifying stroke onset times accurately and cost-effectively. The diagnosis and stroke management can be further altered based on the extent and severity of identified cerebral lesions and to potentially predict HT, improving the overall prognosis [83].

Elsaid et al. discuss several methods of imaging that have been used in stroke centers to evaluate a patient's risk of developing life-threatening complications, namely, HT [84]. Computed tomography perfusion studies can specifically quantify certain parameters of cerebral blood volume, flow, and transit time to assess the risk or extent of HT based on extravasation of contrast material, further analyzing the integrity and permeability of the BBB [84]. Jaillard et al. evaluated CT scan imaging and noted the presence of early CT signs predicted HT. In the MAST-E study, Jaillard and team used prior definitions of loss of the density contrast of the lentiform nucleus, loss of the density contrast of the insular ribbon, and hemispheric sulcus effacement, either alone or in association with a hyperdense MCA sign to score the CT scans [28, 85–89]. In this study, 63% on the patients showed early CT signs. Similar to many other studies, early CT signs predicted both HT and symptomatic HT in their multivariate analysis as well [90–93]. Similarly, a meta-analysis by Wen et al. noted that ischemic signs on CT and extent of parenchymal hypoattenuation on baseline CT were associated with increased HT [20, 43].

On MRI imaging, a persistent perfusion deficit at 3 to 6 hours after initial MRI was associated with HT (83% vs. 30%; *p* = 0.03) [94]. A study by Tong et al. showed a significantly lower initial apparent diffusion coefficient (ADC) and persistent hypoperfusion on perfusion-weighted imaging (PWI) were noted in regions of secondary HT [94].

## 11. Reperfusion Therapy: Intra-Arterial Thrombolysis Using r-tPA and Thrombectomy

As ischemic strokes and transient ischemic attacks (TIA) are common, and thrombolytics such as tPA (alteplase) and endovascular interventions are often employed, it is important to discuss the effects of reperfusion therapy on HT. For instance, there is an increased risk of developing adverse effects such as hemorrhage with tPA.

Likewise, reperfusion therapies such as thrombolytic interventions and thrombectomies are also associated with HT [4, 46]. There is growing evidence that tPA has some unintended roles as extracellular protease and signaling molecule which affects brain remodeling [95]. A study by Larrue et al. noted that parenchymal hemorrhage (PH) and symptomatic intracranial hemorrhage (SICH) were associated with r-tPA while HI was not [20]. In animal models, thrombolytic administration was associated with increased HT when hypertension was induced before and after alteplase [13, 61, 96]. In patients treated with alteplase, maintaining the blood pressure below 185/110 mmHg is suggested to decrease the risk of HT [13, 97]. Similarly, studies have noted that the risk of HT increases with each 10 mmHg rise in systolic blood pressure from 140 to 180 mmHg [98]. An elevated systolic blood pressure (SBP) is also associated with worse outcomes and higher risks of HT in patients treated with endovascular thrombectomy (EVT) [13, 99, 100]. EVT has its limitations due to its narrow therapeutic window as well. A study by Maïer et al. on-pulse pressure variability during mechanical thrombectomy looked at 343 patients showed increased 3-month all-cause mortality (OR = 1.37, 95% CI: 1.01–1.85, *p* = 0.04). It was noted to be an independent predictor of worse clinical outcome in AIS patients [101].

Certain other factors such as renal impairment, defined as elevated creatinine, >1.0 mg/dL, or eGFR (eGFR < 60 mL/min per 1.73 3 m2), after tPA is associated with higher risk of symptomatic intracranial hemorrhage [38]. Few other very interesting factors such as increased arterial stiffness and increased globulin level are also associated with HT especially in the setting of tPA. According to a study by Acampa et al., arterial stiffness is a novel independent risk factor for HT. The study evaluated 258 patients with AIS undergoing intravenous thrombolysis or/and mechanical thrombectomy and found an increased risk for HT was associated with higher arterial stiffness especially when ASI is >0.71 [102]. Tocci and Presta also noted similar association between increased arterial stiffness and HT after thrombolysis [103]. Higher globulin level was noted to be associated with higher risk of HT on single factor analysis (*p* < 0.002) and multifactor logistic regression (*p* < 0.001; OR, 1.185; 95% confidence interval (CI), 1.090-1.288) by Xing et al. [85]. Xing proposes that globulin level is an independent risk factor for HT in patients receiving intra-arterial thrombolysis.

## 12. Use of Antiplatelets in Ischemic Stroke and Hemorrhagic Transformation: Mono-Antiplatelet (Aspirin) versus Dual-Antiplatelet (Aspirin plus Clopidogrel)

The effective and safe use of antiplatelet therapy is crucial for secondary prevention following high risk transient ischemic attack or acute minor ischemic stroke to reduce early recurrence. Aspirin is commonly used as a mono-antiplatelet therapy (MAPT) or in conjunction with other antiplatelets as dual antiplatelet therapy (DAPT) to reduce the risk of stroke recurrence.

According to the American Heart Association, aspirin is the most commonly prescribed antiplatelet medication to help prevent ischemic strokes. The results of a large-scale study performed on patients with a prior history of stroke found that aspirin use reduces the risk of stroke recurrence by about twenty to twenty-five percent [21, 104]. Antiplatelets, however, poses a risk for HT.

In a randomized, double-blind, placebo-controlled (POINT) trial conducted by Johnston et al., patients were assigned to DAPT, MAPT, or placebo group [105]. The DAPT group received aspirin and clopidogrel at a loading dose of 600 mg on day 1, then 75 mg per day of clopidogrel and 50-300 mg of aspirin per day for 2 to 90 days; the MAPT group received only aspirin. After day 5, patients received 81 mg of aspirin. They were followed for 90 days, and a time-to-event analysis was conducted [105]. The study suggested that patients with minor ischemic strokes or TIAs that received the DAPT had a lower risk of developing major ischemic events but an increased risk of developing a major hemorrhage than patients in the MAPT group. Of the DAPT group, 23 patients (0.9%) developed a major hemorrhage, whereas in the MAPT group, 10 patients (0.4%) developed a major hemorrhage [105]. The long-term DAPT therapy of 90 days was noted to have added risks and outweigh the benefits of reducing stroke recurrence.

The CHANCE trial by Wang et al. conducted a randomized, double-blind, placebo-controlled trial at 114 centers in China where placebo plus aspirin (75 mg per day for 90 days) with clopidogrel and aspirin (clopidogrel at an initial dose of 300 mg, followed by 75 mg per day for 90 days, plus aspirin at a dose of 75 mg per day for the first 21 days) was evaluated. They looked at 5170 patients who presented with minor ischemic stroke or high-risk TIA within 24 hours and noted that the aspirin and clopidogrel combination is superior to aspirin alone for reducing the risk of stroke in the first 90 days and does not increase the risk of hemorrhage [106].

Pei et al. analyzed the effect of DAPT and MAPT on stroke risk in TIA patients through positive diffusion-weighted imaging (DWI) [107]. Researchers found that TIA patients on MAPT had a higher 90-day stroke risk than patients that used DAPT (23.7% vs. 13.4%, *p* = 0.029). Additionally, patients who were positive DWI had a higher 90-day stroke risk in comparison to negative DWI patients (*p* < 0.001) [107]. This study also suggests that early administration of DAPT in TIA patients is associated with a 46% decrease of stroke risk in DWI positive TIA patients [107].

In a systematic review by Rahman et al., data from ten randomized controlled trials comprising 15434 patients were assessed. The use of MAPT with aspirin and DAPT with aspirin and clopidogrel for short (<1 month), intermediate (<3 months), and long term (>3 months) therapies was compared to determine the optimal therapy duration [71]. Outcomes such as ischemic strokes and major cardiovascular events were quantified and analyzed. Researchers found that short-term (<1 month) DAPT showed a 47% risk reduction of stroke recurrence in comparison to MAPT patients. However, intermediate and long-term DAPT increased the risk of major bleeding [105].

Similarly, a meta-analysis by Hao et al. suggested that DAPT in high-risk TIA patients can have maximized benefits as it reduces the risk of recurrent strokes by 2% [108]. Starting the combination therapy of aspirin and clopidogrel within 24 hours of TIA or AIS onset and discontinuing it by day 10, or no later than day 21 helps in secondary stroke prevention. Additionally, limiting the combination therapy duration to 21 days does not increase the risk of hemorrhagic transformation occurrence [108]. Through various studies and meta-analyses, researchers found that short-term use of DAPT in high-risk TIA patients is effective in secondary stroke prevention in comparison to MAPT. However, long-term DAPT does not show increased effectiveness and may increase the risk of hemorrhagic events.

Very few studies have also looked at high NIHSS or moderate to high severity strokes and the use of antiplatelets. A single-center retrospective cohort study by Khazaal et al. looked at 377 patients with NIHSS > 4 and compared SAPT with DAPT. They noted that symptomatic ICH and serious systemic bleeding were rare in the DAPT group and no major difference between SAPT and DAPT in moderate to severe ischemic stroke patients [109]. However, more randomized trials are needed to confirm these results before it can be implemented in to practice.

The risk for HT is significantly more when antiplatelets and antithrombotic agents are combined. Zheng et al. also examined the effect of DAPT and the occurrence of HT in mice treated with tPA in comparison to the control group, which were not treated with tPA [110]. Middle cerebral artery occlusion (MCAO) was induced, followed by tPA administration in the experimental group. Cerebral infarct sizes and HT were assessed and quantified as well. The study found that mice treated with DAPT and tPA had a significantly increased HT occurrence (*p* = 0.0045) in comparison to mice treated with DAPT and no tPA (*p* = 0.3784). Reduced dosages of tPA in the experimental group of mice did not reduce the occurrence of HT [111]. This suggests that the use of tPA is associated with an increased risk of developing a HT. Several studies continue to further strengthen the notion of promoting individualized patient treatment plans, while considering previously implemented interventions to reduce the risk of secondary strokes and hemorrhagic events in high-risk patients.

Based on many of these studies listed above along with numerous others, the current American Heart Association guidelines recommend DAPT initiation within 24 hours of low NIHSS AIS or high-risk TIA presentation and continuing this combination therapy for 21 days [57, 71, 83, 105, 108]. The use of DAPT is currently not recommended for high NIHSS or large infarction strokes. The use of DAPT after HT is controversial. A retrospective study by Kim et al. on 222 patients noted that the use of antiplatelets after HT was not associated with worsening neurologic status or aggravating HT [112]. However, currently, there are no official guidelines on this topic, and more studies are needed to further investigate the role of antiplatelets after HT.

## 13. Management of Postischemic Hemorrhagic Transformation

Due to the disabling neurological and motor deficits precipitated by strokes and its complications, patients suffer from additional infections and comorbidities, with an increased length of hospital stay [84]. Hemorrhagic complications result in increased morbidity and mortality rates, carrying a higher economic and social burden on the affected family and the healthcare system. The identification of specific predictors can significantly help physicians and healthcare providers reduce the occurrence of HT. Aside from reducing the risk of HT, the management and treatment options for HT remain limited. Current American Heart Association guidelines for HT management are similar to those of an intracerebral hemorrhage (ICH) [14, 104]. Therefore, stringent assessment of risk to benefit ratios when prescribing certain treatment plans remains as a significant factor for stroke patients.

## 14. Discussion

According to the World Health Organization, strokes are currently one among the leading causes of death and disability worldwide, and therefore, it is crucial that effective and safe interventions are implemented to better manage stroke and its complications. The medical and research community has found additional pharmaceutical and surgical treatments for stroke patients over the course of decades of groundbreaking research in stroke pathophysiology, risk factors, and treatment effectiveness.

Our current review of literature highlights risk factors such as demographic factors, history of essential hypertension, diabetes mellitus, ischemic heart disease, and CHF increases the risk and severity of HT [5, 6, 14, 37, 44]. Certain predictors such as hyperglycemia and systolic blood pressure variability during ischemic event, atrial fibrillation, cardioembolic stroke, low total cholesterol and LDL levels, higher NIHSS score, large infarct size, poor collateral circulation, impact of immune response and inflammatory mediators, blood-brain barrier permeability, early imaging findings on CT/MRI, and reperfusion therapy also increase the risk of HT [13, 14, 20, 24, 31, 38–51, 53–60, 69–75, 80–82, 85, 90, 93, 94]. Few interesting factors such as arterial stiffness, globulin levels, higher body temperature during ischemic events, elevated creatinine, electrolyte abnormalities such as hyperkalemia and hypomagnesemia, leukoplakia, and leukoaraiosis also play major roles in HT [76, 77].

Approximately 40% of stroke patients remain on antiplatelet therapy for secondary stroke prevention and its protective effective are well established [110]. The review of recent literature demonstrates that DAPT is highly effective and safe in high-risk TIA and low severity ischemic stroke patients for only a short time [105]. Although aspirin is widely prescribed for use in the treatment of blood clot disorders, scientists must examine and inform the healthcare community about the benefits of dual antiplatelet therapy before doing so. There is a possible increased risk of developing hemorrhagic events with DAPT treatment over a prolonged period of time. A number of research studies have found that DAPT's use of aspirin and clopidogrel, like a 21-day use period, reduces the risk of hemorrhagic transformation [105–108]. Further research is indicated to analyze the effectiveness of DAPT and its optimal therapy duration in high-risk stroke patients. Therefore, healthcare providers should take into consideration the added risks and benefits of DAPT or MAPT in high-risk patients. Through an analysis of previously existing literature, we discussed the efficacy and safety of mono- versus dual-antiplatelet therapy in patients with AISs, especially when it comes to hemorrhagic transformation and its management. The duration of each specific antiplatelet therapy can be modified on the basis of each patient's specific clinical case. Studies presented have also demonstrated that standard approaches to patient management and treatments cannot be used as “one size fits all.” Each patient's treatment plan should be individualized to maximize treatment length and therapeutic effects.

Invasive treatment options include thrombectomy procedures performed using an endovascular approach, but due to its very limited therapeutic window, this treatment option has limited use. Conventional intravenous thrombolytics, such as recombinant tissue plasminogen activator (rt-PA, alteplase), is effective in reducing clot size, but it can lead to hemorrhagic transformation and cause profuse bleeding [4, 46, 85, 96–102].

Patients with ischemic stroke who experience hemorrhagic transformation need to be closely monitored to ensure no neurological deficits are present. Recent studies note the significance of keeping blood pressure within acceptable parameters to avoid potentially deadly complications and ensure successful reperfusion. The level of severity and location of the hemorrhage is the main consideration when treating patients with hemorrhagic transformation. Improving the prognosis of stroke patients by experimenting with gene therapy could help to restore the BBB integrity. Additional research should be done to gain a better understanding of the pathogenesis and intricacies of hemorrhagic transformation, as it has been shown to be a deadly complication in stroke patients who have suffered an ischemic stroke.

## 15. Conclusion

Understanding the development and effective management of HT in AIS patients is complex. A successful approach can lead clinicians to implement individualized treatment plans to address the underlying causes and associated risk factors. Our article highlights recent developments in the identification and evaluation of risks and predictors in ischemic stroke patients in the development of an HT. The current guidelines and approaches for reducing HT and secondary stroke risk suggest that clopidogrel and aspirin should be used for a short period of 21 days in dual-antiplatelet therapy. In addition, it can identify the gravity and the extent of cerebral damage from HT through the use of detailed neuroimages. Reviewing additional risk factors and predictors for each patient individually along with benefits of dual antiplatelet therapy can improve the stroke prognosis globally while reducing complications such as HT.

### 15.1. Limitations

This article has a few limitations, as it is focused only on patients who have suffered from ischemic stroke and are primarily receiving antiplatelet therapies. Study populations should include patients who have undergone endovascular thrombectomies or thrombolytic drugs, such as alteplase, in order to better investigate the risk of developing hemorrhagic transformation and the effectiveness of dual antiplatelet therapy. Patient risks and treatment responsiveness should be examined in longitudinal studies as well. This piece can serve as a good primer on ground-breaking recent discoveries. Further research is suggested in order to gain a better understanding of the process of hemorrhagic transformation and to improve hemorrhagic transformation treatment techniques to benefit patients.

## Data Availability

No data were used in this study.
